# 11. People Living with HIV During the COVID-19 Pandemic: Who Did (or Did Not) Receive Annual Influenza Vaccination?

**DOI:** 10.1093/ofid/ofab466.213

**Published:** 2021-12-04

**Authors:** Deborah A Kahal, Brian Wharton, Christopher James, Richard Caplan, Bincsik K Arlene

**Affiliations:** 1 Christiana Care Health System, Media, Pennsylvania; 2 ChristianaCare, Wilmington, Delaware

## Abstract

**Background:**

Nationally, younger adults and racial minorities have lower levels of influenza vaccination (influenza vaccination = vaccine) than non-Hispanic White adults. During the 2015-16 season, most vaccine decliners in our program were male, black, and 45-66 years of age. As part of a quality improvement (QI) initiative to increase 2020-21 vaccine coverage amongst PLWH, we sought to compare patient characteristics between vaccine recipients and non-recipients.

**Methods:**

Our program cares for 60% of Delawareans with HIV. The largest site in Wilmington was the QI site. IRB exemption was received, and pre-defined sociodemographic and HIV-specific variables were extracted from the EMR and CareWare from 1 Oct 2020 through 31 March 2021. Patient reports of external vaccine required confirmation. All PLWH ≥ 18 years of age, including those newly establishing care, met eligibility criteria. Comparisons between vaccinated and unvaccinated PLWH were performed using Wilcoxon rank sum tests for continuous variables and chi-squared tests for categorical variables. A multivariable logistic regression model, including age, sex, race, insurance, poverty level, HIV status, and virologic suppression, was used to predict vaccine.

**Results:**

780 patients met study inclusion criteria and 86% (667/780) received vaccine. Characteristics of PLWH with and without vaccine are presented in Table 1. Older age, lower HIV viral load, and virologic suppression had a statistically significant (p< 0.05) association with vaccine receipt in unadjusted analysis. Only older age (p< 0.01) was significantly associated with vaccine in logistic regression modeling (Table 2), however this relationship was non-linear.

Table 1. Characteristics of patients living with HIV during the 2020-2021 Influenza vaccination season

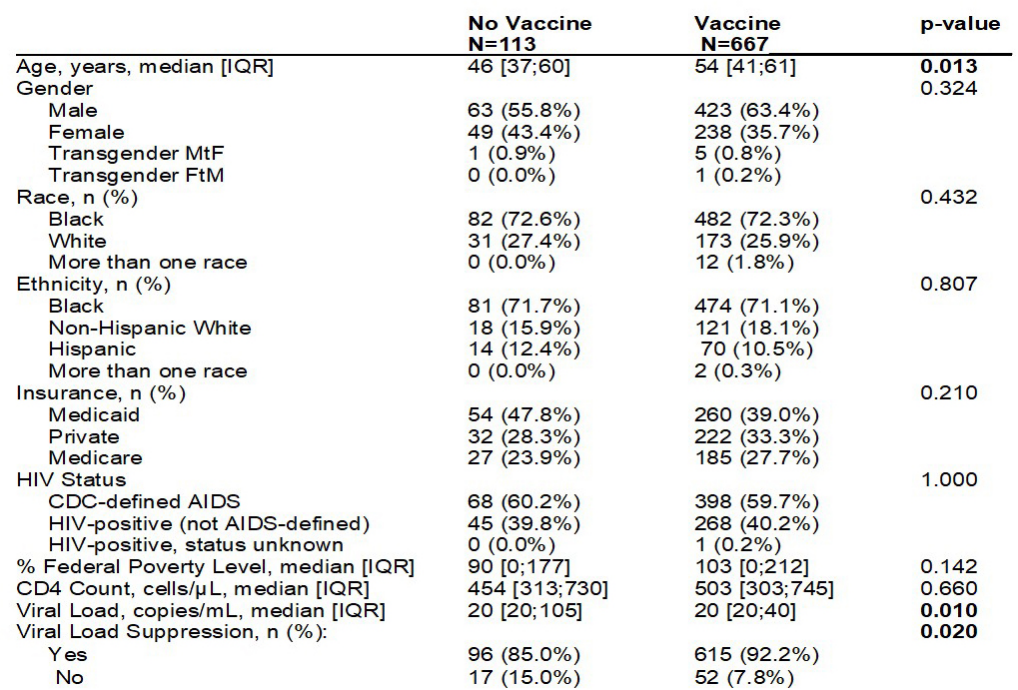

Table 2. Multivariable Analysis of Baseline Characteristics

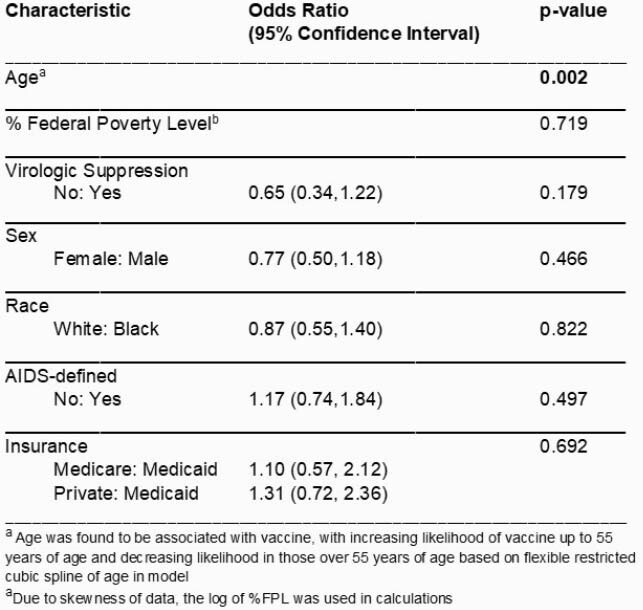

**Conclusion:**

A very high rate of PLWH received vaccine, far exceeding local and national benchmarks, with EMR data unlikely to have fully captured all vaccines. The role of the COVID-19 pandemic in vaccine amongst PLWH is not yet known. While older age was associated with vaccine in adjusted analysis, the number of unvaccinated patients was small, confidence intervals wide, and associations consequently weak. Larger studies are needed to further investigate factors associated with vaccine receipt amongst PLWH.

**Disclosures:**

**Deborah A. Kahal, MD,MPH, FACP**, **Gilead** (Speaker’s Bureau)**Viiv** (Speaker’s Bureau)

